# Supplementary material for: Identification of Molecules from Coffee Silverskin That Suppresses Myostatin Activity and Improves Muscle Mass and Strength in Mice
Source: Molecules. 2021 May 3;26(9):2676. doi: 10.3390/molecules26092676 (PMC8124993; doi:10.3390/molecules26092676)
Supplement: Supplementary file 1 [file molecules-26-02676-s001.zip › molecules-1165435-supplementary.pdf]

Identification of molecules from coffee silverskin that suppresses myostatin activity and improving muscle mass and strength in mice

Jeong Han Kim†[1], Jae Hong Kim†[1], Jun-Pil Jang[2], Jae-Hyuk Jang[3], Deuk-Hee Jin[1], Yong Soo Kim[4] and Hyung-Joo Jin[4*]

.

Figure S1. Western blot result

	1	2	3	4	5	6	7
MSTN (10 nM)	—	+	+	—	—	—	—
GDF 11(10 nM)	—	—	—	+	+	—	—
Activin A(10 nM)	—	—	—	—	—	+	+
CSE (50 ug/ml)	—	—	+	—	+	—	+

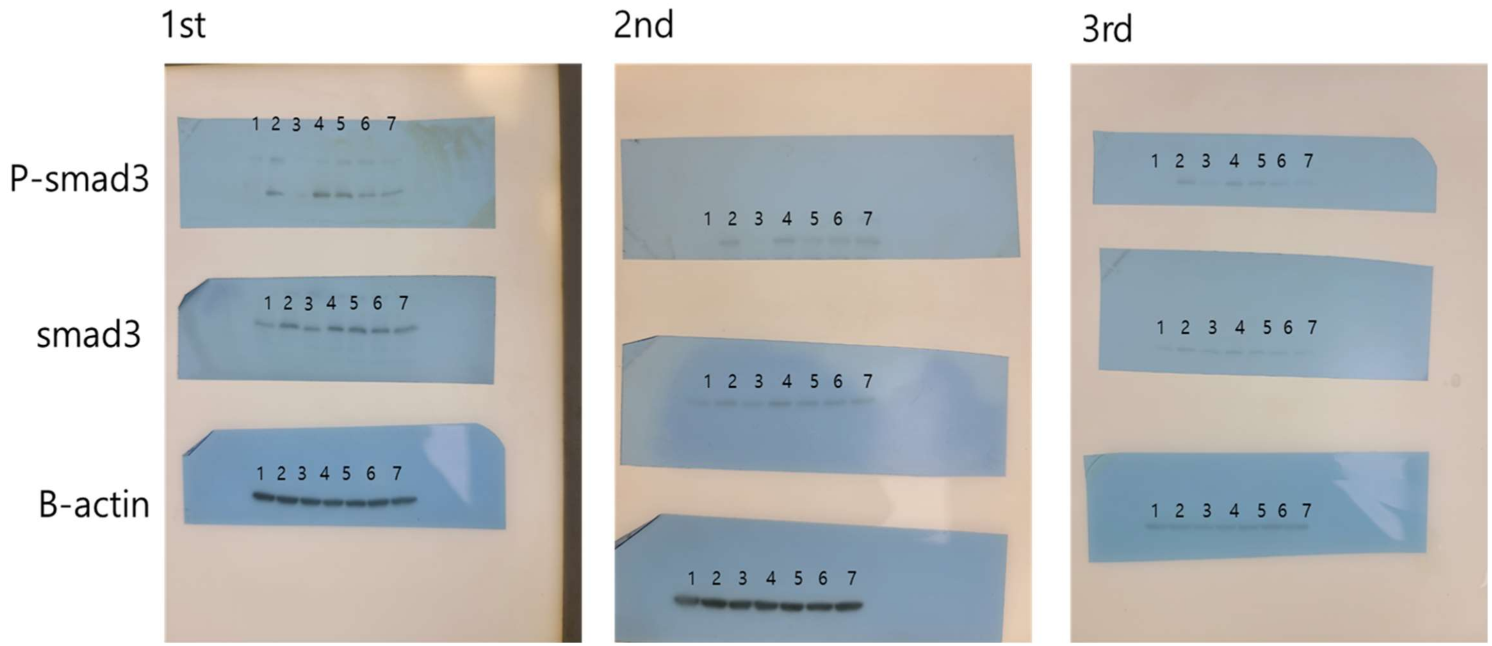

The 1st Western blot result is shown in Fig 3.

Figure S2. qRT-PCR result

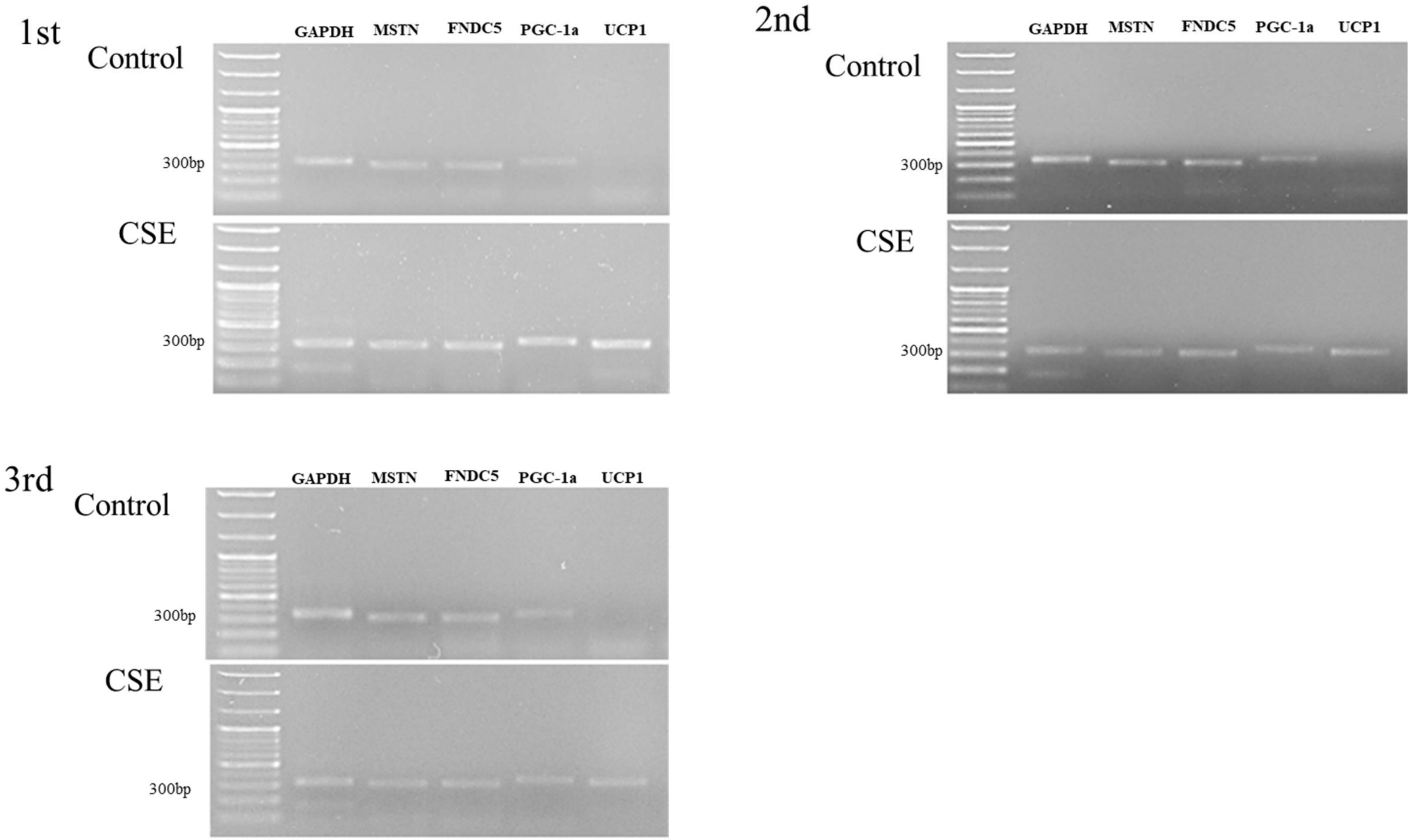

The 1st Electrophoresis result is shown in Fig 5.

Figure S3. NMR spectrum of peak 1

$^{\beta}N$-arachinoyl-5-hydroxytryptamide (C_{20}-5-HT)

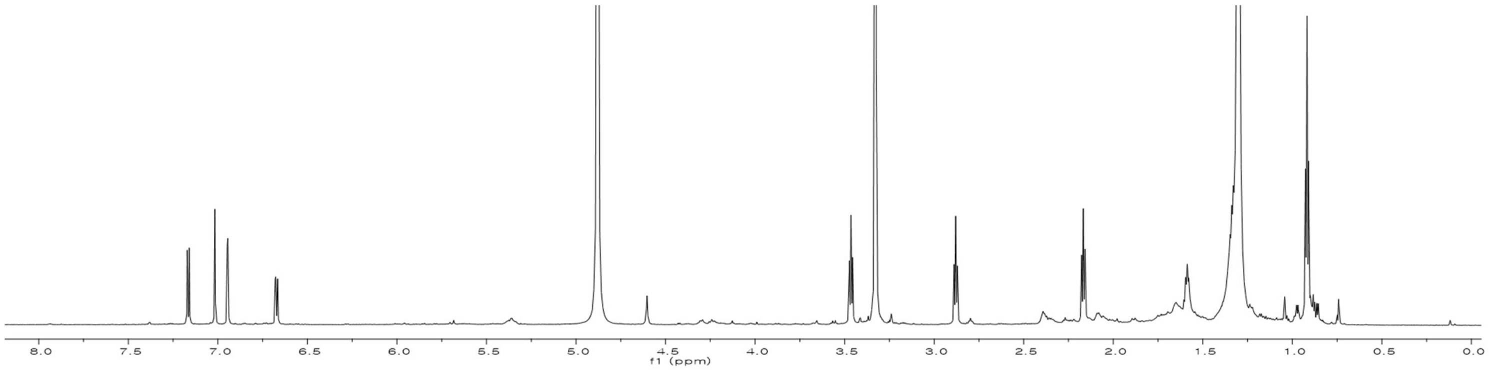

^{1}H-NMR spectrum of SS-P1E (CD_3OD, 800 MHz)

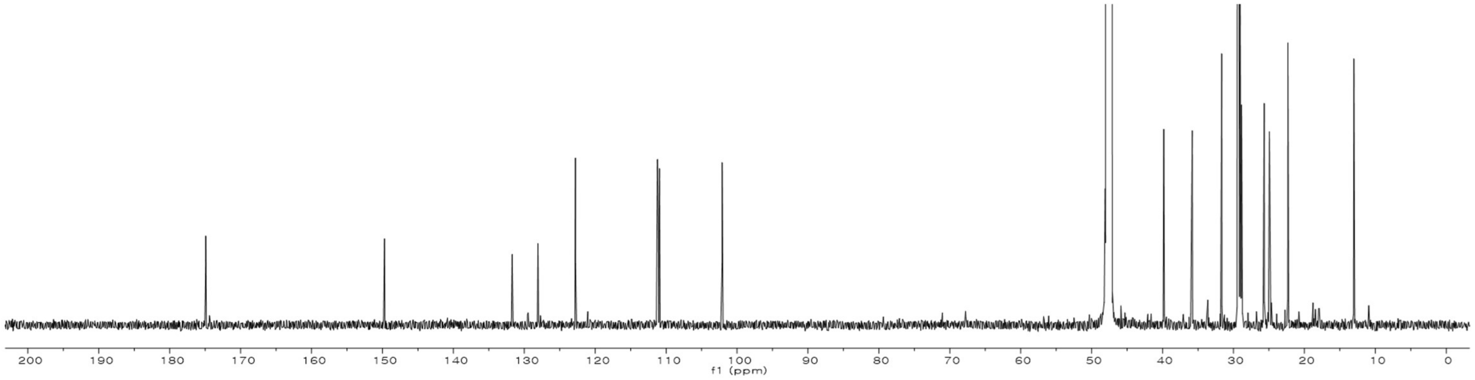

^{13}C-NMR spectrum of SS-P1E (CD_3OD, 200 MHz)

Figure S4. NMR spectrum of peak 2

$^{\beta}$*N*-behenoyl-5-hydroxytryptamide (C_{22}-5-HT)

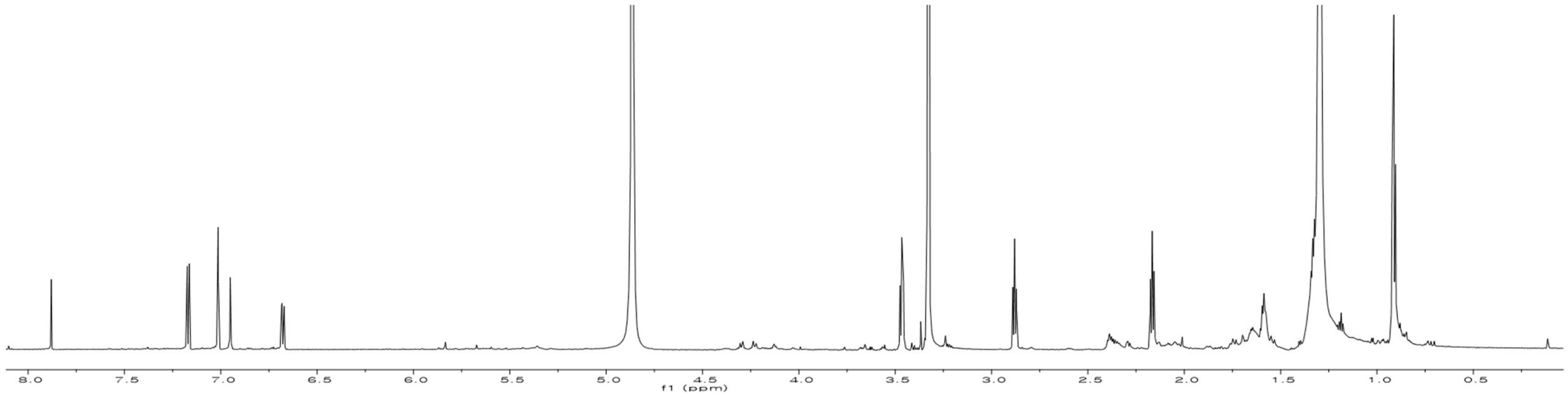

^{1}H-NMR spectrum of SS-P2E (CD_3OD+$CDCl_3$, 800 MHz)

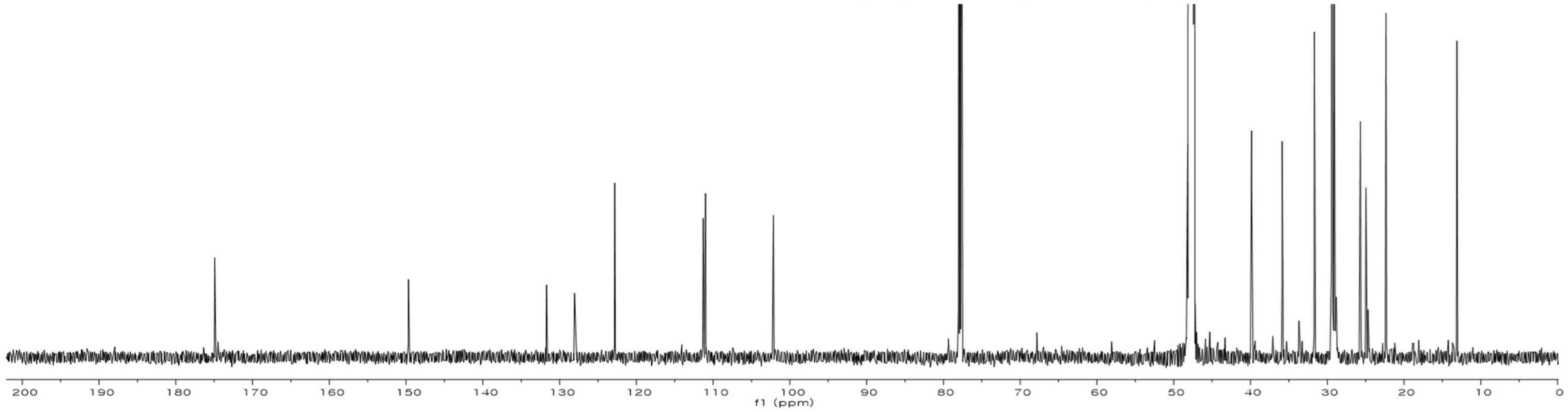

^{13}C-NMR spectrum of SS-P2E (CD_3OD+$CDCl_3$, 200 MHz)
